# Surgical techniques for enhancing postoperative urinary continence in robot-assisted radical prostatectomy: a comprehensive review

**DOI:** 10.1097/JS9.0000000000002414

**Published:** 2025-05-12

**Authors:** Yufei Yu, Robert E. Reiter, Mo Zhang

**Affiliations:** a Department of Urology, The First Hospital of China Medical University, Shenyang, China; b Institute of Urology, China Medical University, Shenyang, China; c Department of Urology, David Geffen School of Medicine, University of California Los Angeles, Los Angeles, California

**Keywords:** prostate cancer, robot-assisted radical prostatectomy, surgery, urinary continence

## Abstract

Prostate cancer (PCa) is one of the most common malignancies affecting the male reproductive system. Robot-assisted radical prostatectomy (RARP) has been a standard treatment for PCa for over 20 years. However, postoperative urinary incontinence remains a frequent complication for patients who undergo RARP. Numerous surgical techniques have been developed to improve postoperative continence recovery, broadly categorized into preservation and reconstruction methods. Preservation techniques include safeguarding the bladder neck, distal urethra, detrusor apron, retropubic space, neurovascular bundles, and controlling the dorsal venous complex. Reconstruction techniques aim to mitigate the impairment of urethral sphincter function caused by surgery. While these approaches substantially enhance post-RARP urinary continence recovery, challenges remain in tailoring surgical plans to individual patient needs. This review explores the application of these representative techniques, discusses their current limitations, and highlights potential directions for future advancement.

## Introduction

Prostate cancer (PCa) is the second most common cancer among men worldwide. In 2022, over 1.46 million new cases of PCa were recorded globally. Robot-assisted radical prostatectomy (RARP) remains a mainstay in the treatment of localized PCa, including some locally advanced cases^[^1^]^. In the United States, the 5-year survival rate for patients with localized PCa undergoing RARP exceeds 95%^[^2^]^. As these survival rates improve, increasing attention has been directed toward surgery-related complications. Patients undergoing RARP exhibit a higher incidence of postoperative erectile dysfunction and urinary incontinence compared to those receiving radiotherapy and some forms of focal therapy (including external beam radiation therapy, brachytherapy, high-intensity focused ultrasound, irreversible electroporation, focal laser ablation, and cryoablation)^[^3–6^]^. Urinary incontinence significantly impairs patients’ quality of life^[^7^]^. In addition to increasing postoperative medical expenses^[^8^]^, urinary incontinence severely restricts patients’ physical and social activities and affects their intimate relationships^[^9,10^]^.

**HIGHLIGHTS**

- This review comprehensively evaluates both preservation and reconstruction surgical techniques aimed at improving urinary continence outcomes following robot-assisted radical prostatectomy.
- It provides a detailed exploration of bladder neck preservation, membranous urethral preservation, detrusor apron preservation, dorsal venous complex control, and nerve-sparing techniques, highlighting their roles in enhancing continence recovery.
- The examination of various reconstruction methods, such as urethral suspension stitches and posterior reconstruction, demonstrates their effectiveness in supporting continence restoration post-surgery.
- The discussion on the potential of augmented reality and predictive modeling emphasizes the move towards individualized surgical plans to further improve continence outcomes and enhance patient quality of life.

It has been over 20 years since Abbou *et al* and Binder Kramer^[^11,12^]^ first attempted laparoscopic radical prostatectomy using a surgical robot. The integration of highly dexterous robotic arms and advanced three-dimensional (3D) high-definition visualization effectively enhances the precision of intraoperative anatomical dissection. By modifying various techniques to preserve or reconstruct anatomical structures, surgeons have made significant advances in postoperative continence recovery^[^13,14^]^. Currently, the widely accepted definition of urinary continence following RARP has been pad-free status (no pad usage per day). Alternative definitions of continence include social continence, characterized by the use of ≤1 pad per day^[^15,16^]^.In this review, we summarize representative surgical techniques developed to date, which can be classified into reconstruction and preservation techniques, and highlight emerging approaches that can continuously improve individual urinary outcomes.

## Literature selection

This review employed a methodical and integrative approach to screen relevant literature, using the PubMed, Embase, and Cochrane Library databases. The following keywords “robotic,” “robot,” “prostatectomy,” “continence,” “preservation,” and “reconstruction” were used in our search. Studies were included if they: (1) were clinical studies or quantitative systematic reviews; (2) focused on urinary continence outcomes following RARP; and (3) were published in English. Studies were excluded if they: (1) focused on nonsurgical interventions; (2) lacked clear methodology or continence data; (3) were unpublished or non-peer-reviewed; or (4) did not have full-text availability. The above search strategy resulted in a comprehensive selection of relevant literature, which formed the basis of our review. An overview of representative techniques focused on post-RARP urinary continence is presented in Table 1.

**Table 1:** Representative techniques focusing on urinary continence after robot-assisted radical prostatectomy

Techniques	First author (year)	Type of study	No. of participants^a^	Median age (years)	Median BMI (kg/ml)	Clinical stage T1/T2/T3 (%)	Gleason score	PSM (%)	Median operative time (min)	Catheter removal time (days)	Continence rate (%)^b^				
											Ins.	1 mo	3 mo	6 mo	12 mo
Hood	Wagaskar *et al*^[^13^]^	One arm	300	64	27	51/35/14	3 + 4	6	169	7	nr	83	91	94^c^	95
Retzius-sparing	Qiu *et al*^[^17^]^	RCT	55	68	24	13/87/0	3 + 4	24	105	7–10	69	nr	88	93	96
ARVUS	Student *et al*^[^18^]^	RCT	32	65	28	nr	nr	13	nr	10	22	63	nr	75	87
FFLU	Schlomm *et al*^[^19^]^	Retrospective	406	63	26	nr	3 + 4	12	nr	nr	nr	nr	nr	nr	95
MAD + NS	Covas Moschovas *et al*^[^20^]^	Retrospective	130	58	27	79/19/2	3 + 4	23	92	nr	nr	nr	92	92	98
EPUP	Nunez Bragayrac *et al*^[^21^]^	Retrospective	48	60	30	nr	3 + 4	19	nr	nr	19	nr	nr	54	nr
Igloo	Fankhauser *et al*^[^22^]^	One arm	13	58	nr	77/23/0	3 + 3	31	200	3	nr	nr	≥92^d^	nr	nr
Super veil	Chang *et al*^[^23^]^	One arm	41	64	nr	nr	nr	15	93	7	24	56	71	85	94
PR	Salazar *et al*^[^24^]^	RCT	81	64	27	78/18/4	3 + 4	nr	nr	5–8	nr	34	59	70	83
PUS	Patel *et al*^[^25^]^	Retrospective	237	60	28	nr	≤6	12	76	5	nr	40	93	98	98

BMI, body mass index; PSM, positive surgical margin rate; mo, month; nr, not reported; RCT, randomized controlled trial; ARVUS, advanced reconstruction of vesicourethral support; FFLU, full functional-length urethral sphincter; NS, nerve-sparing; MAD, modified apical dissection; EPUP, extended prostatic urethral preservation; PR, posterior reconstruction; PUS, periurethral suspension.

All numbers in this table are rounded to the nearest whole number.

^a^ The number of participants enrolled in the experimental group.

^b^ Continence is defined as 0 pads per day.

^c^ Data at 24 weeks.

^d^ Data at 6 weeks.

## Techniques for preservation

### Bladder neck preservation

The aim of bladder neck preservation (BNP) is to keep the proximal part of the lissosphincter as intact as possible. By isolating the lissosphincter from the surrounding tissue first, the surgeon can precisely identify the transection position. Then, the urethra can be transected and subsequently anastomosed at an appropriate diameter. Evidence supporting the efficacy of BNP has been accumulating, with a randomized controlled trial of 199 participants showing continuous improvement in continence over 4 years^[^26,27^]^. One meta-analysis conducted by Kim *et al*^[^28^]^ revealed a better continence rate in the BNP group at 3–4 months, while the outcomes at 12 and 24 months showed a significant superiority in terms of urinary continence. Another meta-analysis reviewed the data from 2284 total participants included in 13 studies and similarly concluded that BNP could accelerate continence recovery rate in and after 12 months^[^29^]^. However, it is worth noting that BNP may increase the risk of positive surgical margins (PSMs), as highlighted by Bellangino *et al*^[^30^]^ in their review of 15 studies, underscoring the need for careful patient selection and meticulous surgical technique.

### Membranous urethra preservation

**Figure 1.**
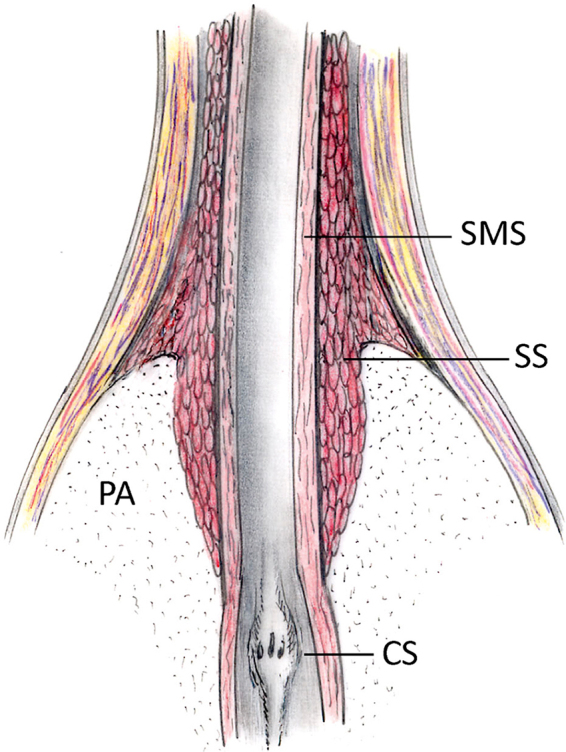
Transversal section of the prostatic apex shows the distribution of striated sphincter^[^19^]^. CS, colliculus seminalis; PA, prostatic apex; SMS, smooth muscle sphincter; SS, striated sphincter (rhabdosphincter).

**Figure 2.**
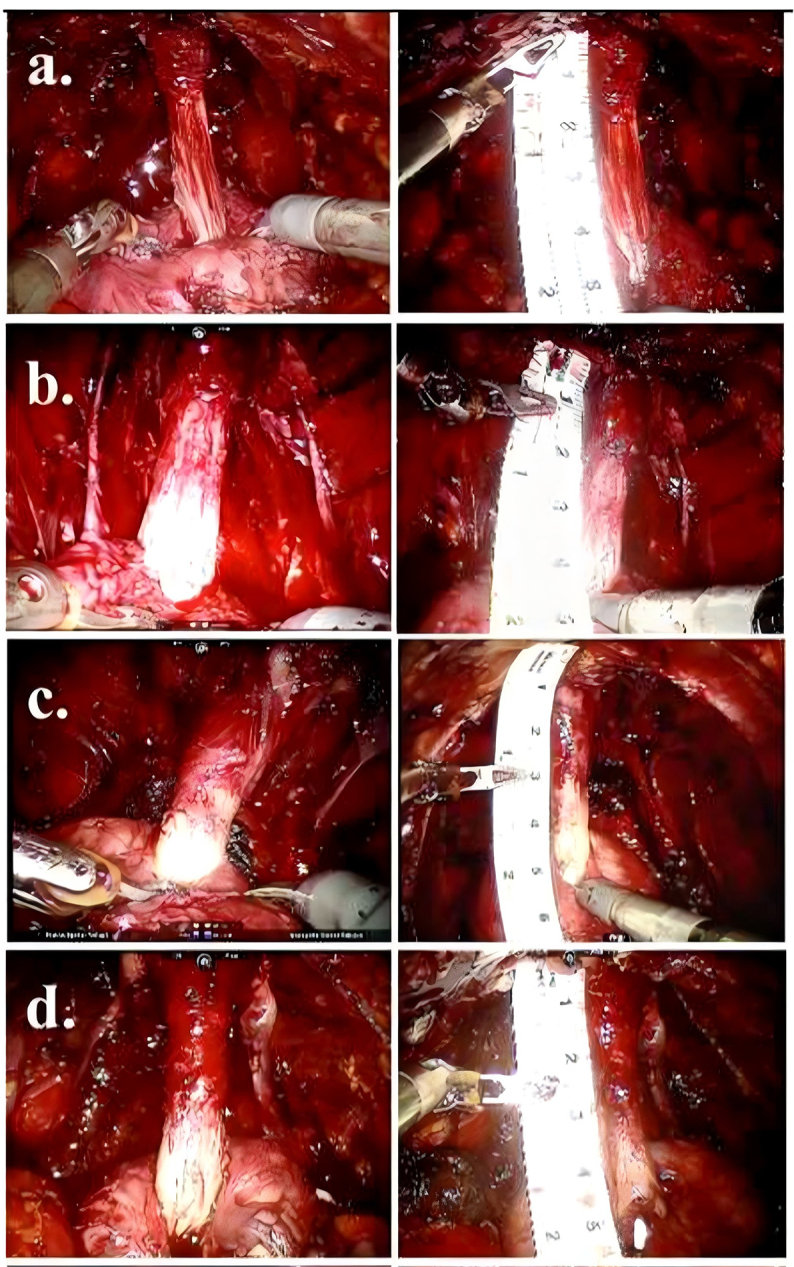
Representative images of urethras preserved during extended prostatic urethral preservation (EPUP) procedures^[^21^]^.

The preoperative membranous urethral length (MUL), as delineated by magnetic resonance imaging (MRI) from the prostatic apex to the penile bulb, has demonstrated a significant positive correlation with both short- and long-term postoperative urinary continence outcomes^[^31^]^. The traditionally conceived external urethral sphincter comprises both voluntary striated and involuntary smooth muscles, surrounding and embracing the prostatic apex^[^19^]^ (Fig. 1). Maximizing the preservation of the membranous urethra contributes to the complete retention of this structure, potentially enhancing urinary continence. When the residual MUL measured by MRI is shorter than 13 mm or MUL decreases by over 6%, the cumulative incidence of postoperative urinary incontinence appears to be higher. In another study, a cutoff point of 16 mm was established based on social continence recovery in 12 months^[^32^]^, providing a guideline for the optimal length when preserving the urethra. When aiming to preserve the distal urethra, surgeons meticulously incise the rhabdosphincter, followed by the circular smooth muscle sphincter, and subsequently the longitudinal smooth muscle sphincter, ensuring clear and precise visualization throughout the procedure^[^33–37^]^. Recently, Kauffman *et al* introduced an aggressive technique referred to as extended prostatic urethral preservation (EPUP) to preserve the longitudinal sphincter. After preserving the external sphincter, they dissected the prostatic urethra along the longitudinal muscular fibers of urethral smooth muscle from the prostate apex^[^21^]^ (Fig. 2). As the entirety of longitudinal sphincter was almost completely preserved, the EPUP group showed significantly better social continence rates at 7 weeks, while the PSM rates were similar between the two groups. Despite the promising urinary continence outcomes, variability in surgeon skill across groups may introduce potential bias, necessitating further validation of the results.

### Dorsal venous complex control

**Figure 3.**
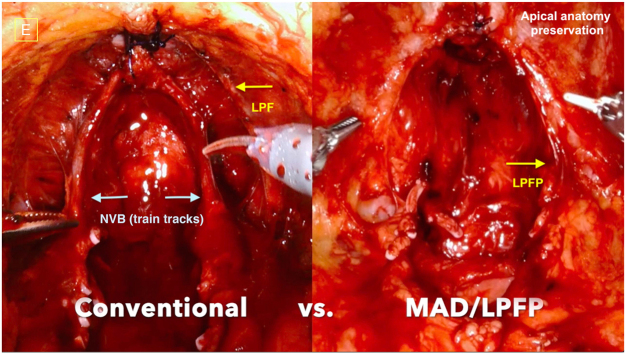
Comparison of the final aspects between conventional RARP and modified apical dissection technique^[^20^]^. LPF, lateral pelvic fascia; LPFP, lateral pelvic fascia preserved; NVB, neurovascular bundles; MAD, modified apical dissection.

Due to the mixed histological composition of the anterior fibromuscular stroma (AFMS) and detrusor apron, specific stratification in the ventral aspect of the prostate is more challenging to discern^[^13^]^. Notably, closer to the apex, potential bleeding from the dorsal venous complex (DVC) further exacerbates the difficulty of achieving clear dissection. The conventional approach to DVC control, involving pre-dissection suturing and ligation, may compromise the integrity of the external sphincter and disrupt anatomical planes, thereby elevating the risk of PSM and complicating membranous urethral preservation^[^34^]^. Lei *et al*^[^38^]^ proposed a technique that involved the initial athermal dissection of the DVC, followed by selective ligation of bleeding venous sinuses. This approach allows for the more precise identification of the anterior surface and the apex of the prostate, free from the interference of preplaced ligatures or sutures during dissection. Among all factors included, age, race, and Gleason score, identified as significant confounders, had been adjusted with stepwise logistic and linear regression models in the study. Their results demonstrated that the approach was associated with higher pad-free continence rate 5 months postoperatively compared to early ligation, although no significant differences were observed in long-term continence. More recent refinements involve dissecting beneath the DVC, thus better preserving it and surrounding structures^[^20,39^]^ (Fig. 3). While some bleeding may occur, this technique allows for the controlled use of thermal devices, minimizing thermal injury to nerves. According to a retrospective study conducted by Covas Moschovas *et al*^[^20^]^, which utilized propensity score matching and strict patient selection to minimize potential confounding and bias, DVC preservation effectively improved the social continence rate within 3 months postoperatively, compared to periurethral suspension. More importantly, studies involving the above technical modifications have not reported any increase in PSMs.

### Detrusor apron preservation

**Figure 4.**
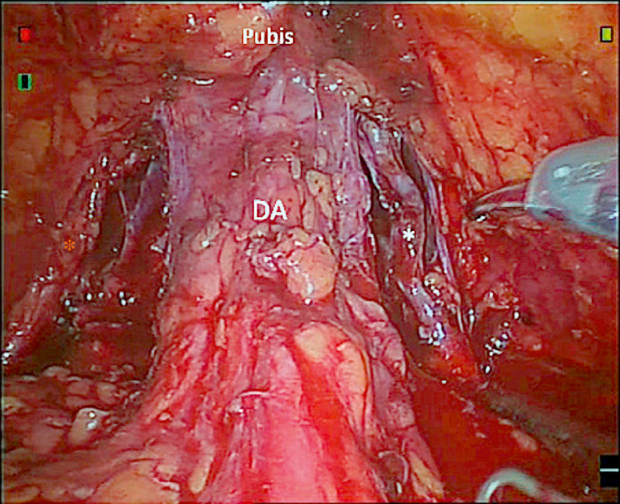
The final aspect of detrusor apron preservation from the anterior approach^[^41^]^. DA, detrusor apron; *accessory pudendal artery.

The outer straight fibers of the detrusor extend beyond the bladder neck and form an apron-like structure covering the anterior surface of the prostate. This apron eventually forms puboprostatic ligaments and attaches to the pubic bone^[^40^]^. In most RARP scenarios, surgeons inevitably transect this structure from the anterior prior to bladder neck transection. Although no studies have elucidated the mechanism of the detrusor apron in urinary continence, preserving this structure at least allows for the retention of surrounding retropubic tissues, which may therefore aid in the recovery of postoperative urinary continence. Tewari *et al* described the “hood” technique that preserves the anatomical structures ventrally, dorsally, and bilaterally in a continuous barrel shape, thereby maintaining the main portion of the detrusor apron and finally achieving an 83% complete continence rate at 5 weeks postoperatively^[^13^]^. Asimakopoulos *et al*^[^41^]^ isolated the prostate from both lateral aspects after dividing the detrusor apron and endopelvic fascia via an anterior approach during RARP to better preserve the detrusor apron as an intact structure (Fig. 4). Their results showed that all 30 participants achieved complete urinary continence within 1 month. Recently, Fankhauser *et al*^[^22^]^ reported a novel “Igloo” technique in which the peritoneum was opened unilaterally to the medial umbilical ligament. The entire process of prostatectomy and subsequent anastomosis was completed from a lateral perspective, avoiding the separation of the detrusor apron from the ventral structures. The median urine loss for 13 participants within the first day after catheter removal was 4 (2–10) g, while PSM was reported in 4 (31%) participants. It is important to emphasize that these techniques have only been evaluated in small single-arm studies involving primarily low- to intermediate-risk groups, utilization of these techniques in high-risk PCa cases requires particular caution.

### Retzius-sparing technique

**Figure 5.**
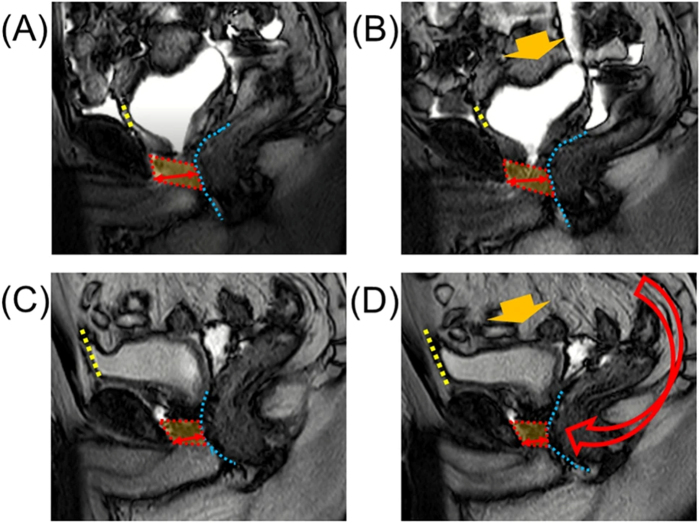
Dynamic mid-sagittal magnetic resonance imaging (MRI) after conventional robot-assisted radical prostatectomy^[^42^]^: at rest (A) and with abdominal pressure (B). Dynamic mid-sagittal MRI after Retzius-sparing RARP: at rest (C) and with abdominal pressure (D). The external urethral sphincter (box surrounded by the red dashed line) was pressed forward by the rectus wall (blue dashed line) under with abdominal pressure (orange arrow).

Conventional RARP (C-RARP) is performed using a retropubic approach, which inevitably causes trauma to the structures anterior to the bladder, thus disrupting the connection between the bladder and abdominal wall. The Retzius-sparing technique, which involves incising the peritoneum at the rectovesical pouch, completes the prostatectomy and anastomosis in a posterior-to-anterior direction, thereby preserving retropubic structures and stabilizing the position of continence-related muscles. Additionally, the postoperative anatomical characteristics may allow the abdominal pressure to contribute to the closing of the urethra. Postoperative MRI scans demonstrated that in patients who underwent C-RARP, the sphincter is pushed dorsally to the soft rectum under temporarily increased abdominal pressure, which makes it harder to close. In contrast, the external urethral sphincter is pushed anteriorly to the pubic bone in Retzius-sparing RARP (RS-RARP) cases (Fig. 5)^[^42^]^. Moreover, the fixation of the anterior bladder wall after RS-RARP facilitates puborectalis contraction, thus to enhance continence recovery^[^43–45^]^. A recent meta-analysis showed significantly better pad-free continence at 1 and 3 months in the RS-RARP group relative to the C-RARP group after reviewing 17 studies. However, PSM rates tended to be higher following RS-RARP for both pT2 and pT3 cases, although the differences were not statistically significant^[^46^]^. In the studies focusing on high-risk PCa patient groups, the PSM rate of RS-RARP reached approximately 30%^[^47,48^]^, indicating that this technique should be employed with particular caution in patients with anteriorly located lesions and high-risk features, especially by surgeons in the early phase of their learning curve. Furthermore, RS-RARP is more technically challenging, especially in the process of urethrovesical anastomosis. Sood *et al*^[^49^]^ recently reported a hybrid operation combining aspects of the anterior and posterior approaches wherein the main procedure was operated anterior to the rectum, while the anastomosis was conducted in the retropubic space with limited trauma. This novel technology achieved equivalent pad-free continence compared with RS-RARP in a small sample size (12 participants in the hybrid group).

### Nerve-sparing techniques

The conventional understanding is that the external urethral sphincter is solely innervated by somatic nerves. Consequently, initially developed nerve-sparing (NS) techniques primarily focused on enhancing postoperative potency outcomes rather than urinary continence. However, a recent study has revealed that the external sphincter is also partially innervated by autonomic nerves running through the neurovascular bundles (NVBs)^[^50^]^. This novel insight aligns with previous findings that the stimulation of NVBs can lead to a rise in intraurethral pressure^[^51^]^ and also provides a theoretical foundation for the application of NS techniques as a means of improving urinary continence. Increasing evidence has suggested that the periprostatic nerves distribute in a layer between the prostatic fascia, pelvic fascia, and the Denonvilliers’ fascia, rather than as a pair of bundles^[^52,53^]^. A recent meta-analysis conducted by Xiang *et al*^[^54^]^, encompassing 26 961 participants, demonstrated that bilateral NS techniques yielded superior urinary continence outcomes as compared to unilateral approaches over a 24-month follow-up period. To better elucidate the concept of NS techniques, this review will focus on two representative approaches.

#### Veil of Aphrodite and super veil

The veil technique was first developed by Menon *et al*^[^55^]^, with a primary focus on potency recovery. The nerve network was preserved along with the outer prostatic fascia, whereas the sector between 1 and 11 o’clock was not preserved due to firm connections within the anterior layers that made it difficult to separate them clearly. In their cohort of over 2500 patients, around 75% of patients achieved complete continence within 3 months. Then, they modified this technique with extra sharp dissection in the anterior sector, simultaneously preserving the nerves between 1 o’clock and 11 o’clock along with the puboprostatic ligaments and DVC, also known as the “super veil” technique^[^56^]^. In a retrospective, single-arm study, Ren *et al* published their outcomes from the super veil technique in 41 patients, who achieved pad-free continence rates of 56.1%, 70.7%, 84.6%, and 94.4% at 1, 3, 6, and 12 months, respectively^[^23^]^.

#### Anatomical grading NS

**Figure 6.**
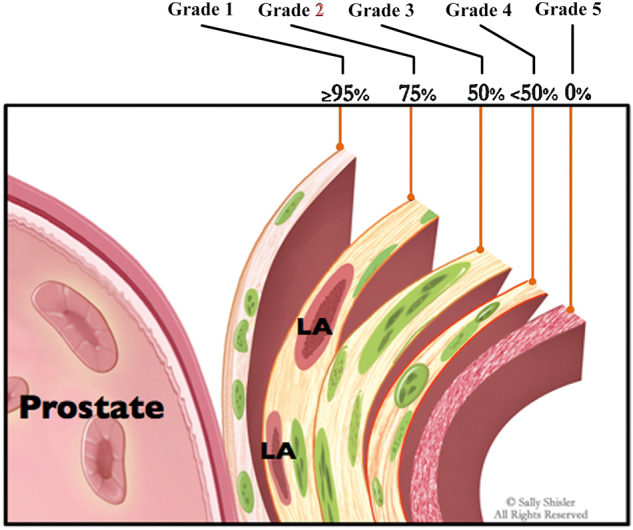
A schematic diagram demonstrating the graded nerve-sparing technique^[^58^]^. Adapted from the work of Sally Shisler. LA, landmark artery.

Capsular arteries consist of a network of arteries originating from the prostatic artery. This vascular network delineates the lateral margin of the prostate. Patel *et al*^[^57^]^ initially noted that these arteries can be used as landmarks for identifying NVBs. They established an anatomic grading retrograde NS technique to standardize this process (Table 2 and Fig. 6)^[^58^]^. This technique led to excellent urinary continence outcomes when combined with several other techniques. Coelho *et al*^[^39^]^ employed a combination of graded NS, maximization of urethral stump preservation, endopelvic fascia and periprostatic collar reconstruction, modified posterior reconstruction, and anterior suspension stitch. Consequently, among 128 patients treated in their study, 85.9% achieved pad-free continence immediately after catheter removal at 7 days post-operation, and 98.4% recovered continence after 1 year of recovery.

**Table 2:** The extent of preservation in anatomical grading nerve-sparing

Grades	Operation procedure	Preservation extent
Grade 5	After the incision of the pelvic fascia, the landmark artery is identified and used to delineate the course of the ipsilateral NVB. The dissection is conducted bluntly alongside the margin of prostate fascia, median to the landmark artery. The plane should consist of avascular areolar tissue.	>95%
Grade 4	The dissection is conducted sharply between the prostate and the landmark artery. A strip of fat without arterial vessels is preserved with the prostate.	75%
Grade 3	The dissection plane is developed at the lateral aspect of the landmark artery. A strip of fat with the capsular artery on the top is preserved with the prostate.	50%
Grade 2	The dissection is performed several millimeters lateral to the landmark artery, and a thick strip of fat is left over the prostate with embedded arteries.	<50%
Grade 1	NVBs are not preserved.	0%

## Techniques for reconstruction

### Urethral suspension stitch

The puboperinealis muscle (PPM), which is the anteromedial part of the levator ani muscle, originates from the pubic bone and runs along both sides of the urethra. During the radical prostatectomy, this muscle can be weakened resulting from damage to the fascia, muscle fibers, or nerve innervation^[^59,60^]^. The purpose of urethral suspension techniques is to alleviate the resistance encountered during the contraction of the PPM. Periurethral urethral suspension^[^25^]^, also known as anterior urethral suspension, was first introduced in the context of RARP by Patel *et al.* To perform the periurethral suspension stitch, the surgeon needs to ligate the DVC and then pass the needle through the periosteum of symphysis pubis to form a loop between the DVC and symphysis pubis under tension. Compared with standard suturing of the DVC, the periurethral suspension stitch group showed a higher social continence rate at 3 months^[^61^]^. A meta-analysis drew similar conclusions when evaluating the evaluation of periurethral stitch technique, which exhibited a positive impact on early continence at 28–42 days^[^62^]^. Cadeddu *et al* have proposed a posterior urethral suspension method. Briefly, for this technique, the rhabdosphincter was pulled toward the pubic bone by the sutures fixed at its posterolateral part on both sides^[^63^]^. In a retrospective trial of 105 participants, the RS-RARP and posterior urethral suspension groups demonstrated comparable urinary outcomes^[^64^]^. However, baseline differences such as a higher proportion of high-risk patients and Gleason scores in the suspension group may affect the results, despite adjustments with multivariable logistic regression. Recently, Leslie *et al*^[^65^]^ introduced a “RoboSling” technique, which utilizes a hammock-like autologous fascial flap to reposition the rhabdosphincter toward the pectineal ligament. Using a prospective non-randomized design, their results demonstrated significantly improved continence rates in the RoboSling group at 3 and 12 months postoperatively.

### Posterior reconstruction

The rectourethral muscle (RUM) lies between the rhabdosphincter and rectum, and it contributes to the closing of the urethra by the rhabdosphincter and PPM aforementioned. The two-layer posterior reconstruction technique starts by suturing the Denonvilliers’ fascia to the dorsal median raphe to stimulate a strong RUM. The needle is then inserted 1–2 cm cranially and posteriorly to the bladder neck^[^66^]^. Besides, all sutures are tightened to relieve the tension of subsequent anastomosis. Regis *et al*^[^67^]^ found that urethrovesical anastomosis tends to be higher in patients who underwent posterior reconstruction than in the control group, potentially altering the angle between the urethra and bladder, thereby improving urinary continence. A pooled analysis also demonstrated that posterior reconstruction is positively associated with both short- and long-term complete continence^[^62^]^. However, this impact on long-term continence was not consistently observed across various studies^[^24^]^.

### Combined reconstruction

To enhance the adjacent structures surrounding the anastomosis, a combination of diverse reconstruction techniques has been employed. Classical posterior reconstruction is typically a component of total reconstruction when dealing with the posterior structures^[^68,69^]^. Student *et al*^[^18^]^ proposed a unique posterior reconstruction technique by suturing the dorsal median raphe, the Denonvilliers’ fascia, the retrotrigonal layer, and bilateral levator ani muscles. The major differences among these techniques lie in the methods taken to handle the anterior and lateral structures. This may include the preservation of puboprostatic ligaments and arcus tendinei^[^68^]^. Some surgeons choose to implement the periurethral suspension stitch technique as the anterior component of this approach^[^70^]^, while others favor anterior pelvic fascia suturing to provide anterior fixation^[^71^]^. There are also anterior reconstruction techniques that attempt to conduct anterolateral reconstruction by suturing the bladder wall to anterolateral pelvic fascia^[^18^]^. Most existing studies on combined reconstruction have demonstrated advantages in urinary continence without compromising oncological outcomes. These techniques, which primarily focus on repairing the pelvic fascia and muscles, are presumed to be favorable for high-risk patients, especially those with severe pelvic floor damage. However, due to the intricate and varied nature of the specific techniques employed for such combined reconstruction, there remains a lack of repeated validation for some particular methods.

### The application of human cells, tissues, and cellular and tissue-based products in nerve reconstruction

Even in NS RARP procedures, thermal coagulation and intraoperative traction may still potentially damage the neural tissues. In recent years, there has been a trend toward utilizing human cells, tissues, and cellular and tissue-based products (HCT/Ps) to achieve reconstruction of wounded nerves. Notably, human placental derivative allografts, such as dehydrated amnion/chorion membrane (dHACM), cryopreserved umbilical cord (Cryo UC), and cryopreserved umbilical cord and amniotic membrane (Cryo UCAM) materials, have been employed to wrap preserved NVBs. These allografts create an optimal cytokine and biochemical environment that supports peripheral nerve regeneration^[^72,73^]^. Currently, three studies have reported urinary continence outcomes after applying human placental derivative allografts in NS-RARP. Two propensity score-matched studies suggested that dHACM facilitated faster recovery of pad-free continence in patients^[^74^]^, while the use of Cryo UC and Cryo UCAM demonstrated comparable efficacy^[^75^]^. Furthermore, the study conducted by Ahmed *et al*^[^76^]^ supported the application of Cryo UC allografting to improve the early and long-term recovery of social continence. Notably, patients with risk factors of incontinence, such as advanced age and obesity, were observed to benefit more from Cryo UC application. Existing studies have not reported on the adverse effects of these aforementioned materials on biochemical recurrence.

## Limitations and future perspectives

Despite our comprehensive literature search and analysis, several limitations warrant mention. First, the heterogeneity in study designs, patient populations, and outcome measurements complicates direct comparisons and may limit the generalizability of these findings. Second, variations in surgeon skill across different centers could significantly influence postoperative outcomes, making it challenging to distinguish the effect of surgical techniques alone. Lastly, the potential for reporting bias should be acknowledged, as observational studies and retrospective analyses may selectively report favorable outcomes, thereby distorting the results. Further standardized, well-powered prospective studies with larger sample sizes are still needed to address these limitations and provide more definitive conclusions.

With the rapid progress in intraoperative navigation, artificial intelligence, and multimodal modeling, new opportunities have emerged to improve postoperative urinary continence and deliver personalized treatment strategies for PCa patients. Of note, recent advances in 3D reconstruction from digitalized images now make it possible to provide intraoperative surgical navigation. By overlapping a 3D model over the real anatomy, augmented reality (AR) can assist surgeons in identifying the relationship between NVBs and lesions, thereby adjusting the surgical plan. Schiavina *et al*^[^77^]^ demonstrated that AR led to NS plan modifications in 38.5% of cases, with 70% of these changes favoring more aggressive approaches, such as adopting NS or expanding from unilateral to bilateral NS, which may potentially improve urinary continence outcomes. Similarly, a prospective study showed that patients undergoing AR-assisted RARP achieved a social continence rate of 90% in 1 month^[^78^]^, surpassing the control group. In addition, the surgeons’ inability to accurately assess the postoperative urinary continence outcomes and the specific benefits of individual surgical techniques makes it challenging to provide personalized combinations of techniques. Currently, there have been several attempts to establish post-prostatectomy continence models^[^79–81^]^. Tillier *et al*^[^82^]^ found that informing patients about the predicted postoperative continence outcomes would influence their choice of treatment strategy. By incorporating different techniques as variables into predictive models, there is an opportunity to offer detailed guidance regarding individual surgical approaches for PCa patients.

## Conclusions

A better understanding of the sophisticated anatomy of periprostatic structures has led to the continuous introduction of new surgical techniques to RARP and supporting efforts. While the current techniques appear encouraging with respect to their impact on postoperative urinary continence recovery, there are still some limitations that will restrict their benefits to patients. In the near future, innovations in imaging and robot-assisted surgical platforms, quantitative assessment of technical benefits, and individualized risk assessment for urinary incontinence will facilitate the formulation of more precise and personalized surgical procedures. This is expected to further improve urinary continence post-RARP, ensuring a better quality of life for PCa patients.

## Data Availability

The data in this review are not sensitive in nature and are accessible in the public domain. The data are therefore available and not of a confidential nature.
